# Chromosomal-Level Reference Genome for the Chinese Endemic Pygmy Grasshopper, *Zhengitettix transpicula*, Sheds Light on Tetrigidae Evolution and Advancing Conservation Efforts

**DOI:** 10.3390/insects15040223

**Published:** 2024-03-25

**Authors:** De-Long Guan, Ya-Zhen Chen, Ying-Can Qin, Xiao-Dong Li, Wei-An Deng

**Affiliations:** 1 Key Laboratory of Ecology of Rare and Endangered Species and Environmental Protection, Guangxi Normal University, Ministry of Education, Guilin 541006, China; 2023660006@hcnu.edu.cn (D.-L.G.); tanyingcan1@hcnu.edu.cn (Y.-C.Q.); 2 Guangxi Key Laboratory of Sericulture Ecology and Applied Intelligent Technology, School of Chemistry and Bioengineering, Hechi University, Hechi 546300, China; 2020660058@hcnu.edu.cn

**Keywords:** Tetrigidae, *Zhengitettix transpicula*, rare orthopteran, conservation genomics, chromosomal genome, CYP305m2 gene

## Abstract

**Simple Summary:**

Pygmy grasshoppers (Orthoptera: Tetrigidae) have evaded genomic characterization thus far, impeding their evolutionary and molecular genetic analysis. Here, through high-throughput sequencing, we pioneer the first reference-grade chromosomal assembly for the Chinese endemic pygmy grasshopper, *Zhengitettix transpicula*. This breakthrough genome provides a foundational resource to expedite the conservation of this endangered species and further contribute to primary and comparative research across Tetrigoidea and Orthoptera in general. Our assembly advances best practices for resolving complex genomes in non-model Orthopterans. Annotation identifies an extensive catalog of protein-coding elements, availing access to the functional elements underlying Tetrigidae’s unique traits. Comparative analyses reveal lineage-specific evolutionary trajectories related to ecological specialization, unlocking questions regarding the genetic trade-offs underlying extreme adaptations. Beyond evolution, our work enables crucial genetic investigations assessing diversity and inbreeding, essential for habitat management interventions to prevent extinction. Variability in the critical defensive gene could underlie the inability of Tetrigidae to form a population outbreak. Thus, this timely and high-quality pygmy grasshopper genome is an invaluable blueprint for accelerating genotype-phenotype connections, evolutionary interpretations, and applied conservation for obscure and iconic species facing similar anthropogenic threats in increasingly fragile ecosystems. Overall, our multidimensional genomic resource promises to invigorate diverse research directions and breakthrough findings across Orthoptera biology, genomics, evolution, and conservation.

**Abstract:**

The pygmy grasshopper, *Zhengitettix transpicula*, is a Chinese endemic species with an exceedingly limited distribution and fragile population structure, rendering it vulnerable to extinction. We present a high-continuity, chromosome-scale reference genome assembly to elucidate this species’ distinctive biology and inform conservation. Employing an integrated sequencing approach, we achieved a 970.40 Mb assembly with 96.32% coverage across seven pseudo-chromosomes and impressive continuity (N50 > 220 Mb). Genome annotation achieves identification with 99.2% BUSCO completeness, supporting quality. Comparative analyses with 14 genomes from Orthoptera-facilitated phylogenomics and revealed 549 significantly expanded gene families in *Z. transpicula* associated with metabolism, stress response, and development. However, genomic analysis exposed remarkably low heterozygosity (0.02%), implying a severe genetic bottleneck from small, fragmented populations, characteristic of species vulnerable to extinction from environmental disruptions. Elucidating the genetic basis of population dynamics and specialization provides an imperative guideline for habitat conservation and restoration of this rare organism. Moreover, divergent evolution analysis of the CYP305m2 gene regulating locust aggregation highlighted potential structural and hence functional variations between Acrididae and Tetrigidae. Our chromosomal genomic characterization of *Z. transpicula* advances Orthopteran resources, establishing a framework for evolutionary developmental explorations and applied conservation genomics, reversing the trajectory of this unique grasshopper lineage towards oblivion.

## 1. Introduction

Pygmy grasshoppers (Orthoptera: Tetrigoidea) have emerged as a distinctive lineage of miniature insects, showcasing unique ecological attributes and life-history strategies that stand in stark contrast to their relatives, the locusts, within the same order (Orthoptera) [1,2]. Unlike locusts, which possess enlarged body sizes, exhibit gregarious behavior, undertake long-distance migrations, and have a propensity for outbreaks that have negatively impacted agricultural systems for millennia, pygmy grasshoppers lead a more reticent lifestyle [3,4]. They predominantly reside in the relaxed, dimly lit environments of shaded mountain streams and moist forests, adopting a life of limited flight and dispersal while subsisting on a specialized diet of fungi [1,5,6,7]. These stark disparities provide a compelling comparative framework through which the genetic and genomic underpinnings of such divergent survival strategies and adaptations—forged by dissimilar selective pressures—may be examined [3,8,9]. However, the previous lack of genomic resources for pygmy grasshoppers has hindered a comprehensive exploration of their distinctive biology.

With over 1800 identified species in China, accounting for more than 95% of globally described Tetrigoidea species [2,10,11], this study presents the first whole-genome sequencing endeavor alongside the assembly and annotation of a Chinese endemic pygmy grasshopper species—*Zhengitettix transpicula* [10,11]. This species was specifically selected due to its unique ecological characteristics. The exceedingly small populations and highly restricted distribution of *Z. transpicula*, confined to a singular valley on Mt. Shiwandashan in Guangxi Province—a prosperous bastion of endemic flora and fauna—renders it an emblematic species that reflects the biodiversity and ecological dynamics of this region [10]. According to our intensive field studies, the scarcity of *Z. transpicula* observations in its natural habitat—fewer than two individuals per day—raises concerns about its endangered status and the perilous loss of its genetic diversity. The plight of this rare Orthopteran in the wild presents an invaluable opportunity to explore and conserve rare members of its order. A thorough genomic assessment of its population structure and genetic diversity will provide imperative guidelines for habitat conservation and species revival strategies. These strategies are crucial in countering the ongoing anthropogenic threats confronting *Z. transpicula* and other endemic organisms on Mt. Shiwandashan.

Furthermore, with chromosomal-level genomic assemblies for pygmy grasshoppers being scarce, comparative genomic analysis can unveil the basis of *Z. transpicula*’s distinctive adaptations—which include ecological specialization, flightlessness, population fragility, developmental constraints on body size, and heightened sensitivity to environmental disturbances [10,11]. Its niche-specific ecology, preferring humid mossy mountainous environments and a fungal diet, coupled with its limited flight ability and lower reproductive rates, vividly contrasts with the adaptable and more generalist locust species thriving in grassland ecosystems [4,5,9,10]. Investigating *Z. transpicula*’s genome is poised to shed light on the genetic underpinnings of ecological specialization and the associated evolutionary trade-offs within a range of orthopteran insects [2].

In our study, we employed advanced high-throughput sequencing and genome assembly methodologies—including HIC, HiFi, NGS, and RNA-seq—to facilitate the articulation of a chromosome-level reference genome for *Z. transpicula*. This represents the inaugural genome for this species and genus and is one of the few within the Tetrigidae family. Building upon this foundation, we implemented comprehensive gene annotation and meticulous refinement of coding genes central to crucial biological pathways. Additionally, we conducted molecular evolution and population genetic analyses to discern lineage-specific gene expansions and survey genetic diversity within natural populations. Our multi-omics investigation of the *Z. transpicula* genome garners pioneering insights, highlighting distinctive genetic features of pygmy grasshoppers vis-à-vis locusts and outlining a delicate population structure that necessitates tailored conservation strategies. As the archetypal reference genome for pygmy grasshoppers, this trailblazing effort propels our comprehension of Tetrigoidea genetics and genomics forward, epitomizing the intricate interplay of genomic science in entomology, evolutionary developmental biology, and conservation initiatives.

## 2. Materials and Methods

### 2.1. Specimen Acquisition

*Zhengitettix transpicula* specimens originated from a wild population in Yizhou, Guangxi Province, China, and were cultivated at Hechi University. Fifth-instar nymphs were selectively preserved for extraction of high-purity genomic DNA and RNA. The female bodies, excluding the abdomen, were utilized on the Illumina NovaSeq 6000 platform for pair-end short-read sequencing and the PacBio HIFI platform for whole-genome sequencing. Concomitantly, leg muscle tissues were harvested for transcriptome and Hi-C sequencing. Two distinct *Z. transpicula* samples from the identical population were chosen for transcriptomic analyses. The tissues were cryopreserved at −80 °C pending DNA/RNA extraction.

### 2.2. Genomic and Transcriptomic Sequencing

A total of five female specimens were utilized for sequencing experiments, and muscle tissues were employed. Three individuals were designated for DNA sequencing, while the remaining two were allocated for transcriptome analysis via RNA sequencing. Genomic DNA was extracted from the designated samples using the Qiagen DNA Mini Kit (Qiagen, Hilden, Germany), following the manufacturer’s instructions. The integrity and quality of the isolated DNA were assessed using an Agilent 2100 Bioanalyzer (Agilent Technologies, Waldbronn, Germany) and a Qubit 3.0 Fluorometer (Invitrogen, Beijing, China), with further validation performed through 1% agarose gel electrophoresis.

For whole genome assembly construction, DNA extracted from one female specimen was utilized for PacBio HiFi library preparation using the SMRTbell Template Prep Kit 2.0. The resulting library was sequenced on the third-generation PacBio Sequel II platform, generating ~37.44 GB of long HiFi reads. In parallel, DNA from the remaining two female specimens was pooled, and total genomic DNA was isolated using the Qiagen DNA Mini Kit (Qiagen, Germany). Whole genome next-generation sequencing (NGS) of these two pooled samples was conducted on the Illumina NovaSeq 6000 system (Illumina, San Diego, CA, USA) to generate short reads. Two paired-end libraries with an insert size of 350 bp were constructed using the Illumina NovaSeq XT DNA library construction kit (Illumina, San Diego, CA, USA), producing ~31.91 Gb of re-sequencing data. Furthermore, the pooled DNA from these two female specimens was subjected to Hi-C (High-throughput Chromosome Conformation Capture) sequencing, yielding ~70.48 Gb of data. The Hi-C procedure was performed using the ARIMA-Hi-C kit (ARIMA, San Diego, CA, USA), and the libraries were prepared according to the manufacturer’s instructions, employing the KAPA hyper preparation kit (KAPA, Boston, MA, USA).

For transcriptome analysis, total RNA was isolated from the remaining two female specimens using the Qiagen RNA isolation kit (Qiagen, Hilden, Germany). Complementary DNA (cDNA) libraries were constructed from the purified RNA through reverse transcription. The transcriptome library preparation was carried out using the DNB preparation kit (BGI, Shenzhen, China). The prepared whole genome and transcriptome libraries were subsequently sequenced also on the Illumina NovaSeq 6000 platform, utilizing the prepared cDNA libraries. A total of 5.54 GB raw transcriptomic sequencing data were produced.

### 2.3. Sequencing Data Quality Control

All raw sequencing data from multiple platforms underwent stringent filtering to eliminate low-quality bases and duplicate reads using GAAP v1.0, which implemented both the FastQC and Trimmomatic tools [12]. Adaptors and duplications originating during library construction were deleted from Illumina sequencing reads.

### 2.4. Estimating Genomic Size and Heterozygosity

A genome survey (k-mer analysis) utilizing the filtered Illumina reads evaluated genomic characteristics. The Jellyfish tool from Trinity v2.11.0 software computed the k-mer number and distribution (k = 21) [13]. GenomeScope v2.0 estimated genome size and performed depth frequency distribution analysis [13,14].

### 2.5. Genomic Assembly and Evaluation of Quality

Genomic assembly was performed using Hifiasm v1.8.0 [15]. Our iterative approach involved repeated mapping, assembly, alignment scoring, and polishing. Subsequently, we employed two complementary approaches, CHROMAP v 0.2.4-r467 [16] and YAHS v 1.2a.1 [17], to mount contigs utilizing high-throughput chromosome conformation capture (Hi-C) data. CHROMA utilizes long-range Hi-C contact information to build an initial scaffold for the contigs, while YAHS employs a hierarchical clustering algorithm to cluster contigs based on their Hi-C interaction patterns. Additionally, we applied Juicebox v3.1.4, a visualization and analysis tool, to refine the mounting process and generate the final results. Sequence statistics of raw and chromosome assemblies are shown in Supplementary Table S1. An interactive linkage map of Hi-C data and the pseudo-genomes is provided in Supplementary Figure S1. Genome annotation combined homology-based and ab initio prediction strategies, integrating RepeatModeler v2.0 [18,19], RepeatMasker v4.1.0 [18,20], and MAKER v3.01.03 [21]. Sequence statistics of repetitive elements and protein-coding genes are shown in Supplementary Table S2. Protein-coding gene annotation utilized the MAKER pipeline, incorporating ab initio prediction, homology-based, and transcriptome-based approaches. Homology protein sequences were downloaded from the NCBI assembly database, and all available records under the term “orthoptera” were employed. Raw transcriptomic reads were initially trimmed using fastp v0.23.2 [22], then mapped to the reference to construct into transcripts using Hisat2 v2.2.1 [23]. We employed InterProScan v5.62-94.0 [24], a widely used highly integrated database, to perform functional annotation of all PCGs using the default embedded databases. The functional annotations were provided in Supplementary Table S3. We used BUSCO v5.4.6 (Benchmarking Universal Single-Copy Orthologs) to evaluate the quality and completeness of a genome assembly and associated gene set [25]. Specifically, we utilized the insecta_odb10 database, which comprises a collection of 1367 single-copy orthologous insect genes. A short summary for BUSCO analysis is provided in Supplementary Table S4.

### 2.6. Comparative Genomic Analysis

OrthoFinder v2.3.8 [26] enabled genomic comparisons between the *Z. transpicula* genome and 14 established qualified Orthoptera genomes. The names and accession of these selected species are provided in Supplementary Table S5. To ensure a uniform level of analysis across various species and circumvent the challenges posed by unpublished annotations in multiple genomes, each of the fifteen available orthopteran genomes, including our newly obtained genome of *Z. transpicula*, underwent a comprehensive reannotation process. For this purpose, Miniprot v0.13_r248 software was employed to facilitate high-throughput gene prediction with rigorous accuracy. The protein sequences used for the gene-finding pipeline were derived from our current gene findings in *Z. transpicula* and complemented by the available protein sequence files from six *Schistocerca* genomes accessible via the NCBI. To optimize the analysis and eliminate redundancy, the collective set of protein sequences underwent a clustering process using the CD-HIT tool v4.8.1 [27] at a 75% similarity threshold, effectively merging sequences with considerable homology.

The multiple amino acid sequence alignment generated by Orthofinder was employed to infer the phylogenetic tree. A phylogenetic divergence time tree was built using the MCMCtree of the PAMLX v1.3.1 [28] software, incorporating divergence times from TimeTree (http://timetree.org/, accessed on 17 January 2024), including 104.3 Mya for *L. migratoria* and *S. gregaria*, and 239.3 Mya for *G. bimaculatus* and *M. thalassinum*. CAFE v5.0 [29] detected gene family expansion/contraction. Plots were generated using OmicStudio tools at https://www.omicstudio.cn/tool, accessed on 27 January 2024. Circos v0.69 [30] visualized the circular genome.

## 3. Results

### 3.1. Genome Assembly

As the foundation of our analysis, we constructed the genome assembly of *Z. transpicula*. We initially conducted a genome survey, which estimated the genome size to range from 895.4 Mb to 897.3 Mb. Guided by this survey, the preliminary assembly using PacBio HiFi circular consensus sequencing (CCS) data yielded a total size of 1.08 GB, which was reduced to a clean draft assembly of 993.95 Mb after heterozygosity filtering. This draft assembly consisted of 386 contigs with a contig N50 of 53.76 Mb and a contig N90 of 6.18 Mb, representing exceptionally high and satisfactory contiguity levels. The GC content of the draft assembly was 34.68%.

**Figure 1:**
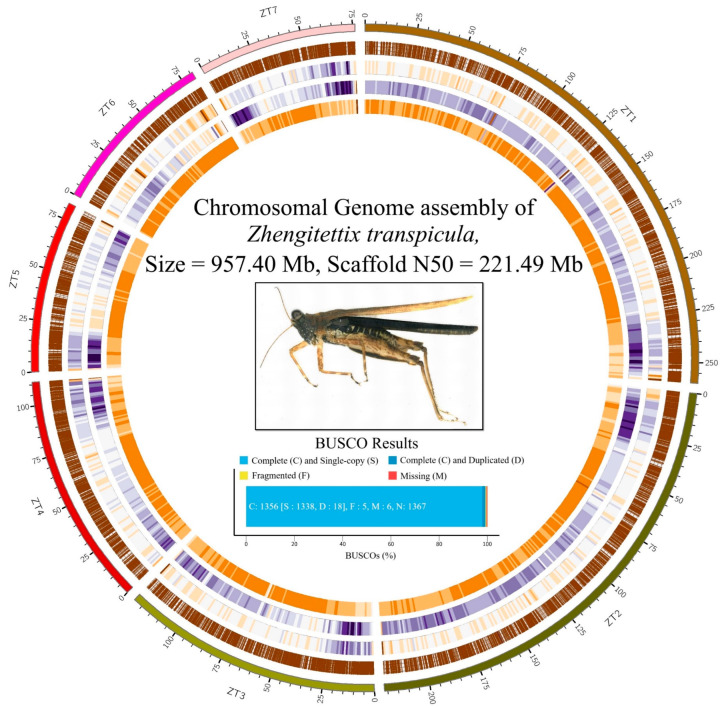
Circos representation of the *Z. transpicula* genome. The layers of the depiction from inside to outside represent the density of GC content, frequency of repeat elements, distribution of variant sites, and gene expressions. The color gradient from light to dark represents the density of different layers, ranging from low to high. Nested within the Circos diagram, a dorsal view of *Z. transpicula* is presented as a species identification. The figure also includes a metric panel illustrating the Benchmarking Universal Single-Copy Orthologs (BUSCO) assessment, which was utilized to estimate the completeness of the chromosomal genome assembly.

Subsequently, we employed Hi-C data to arrange the 54 longest contigs into seven large genome scaffolds, often referred to as pseudo-chromosomes. The longest of these scaffolds reached a size of 259.85 Mb, while the shortest measured 76.24 Mb. The combined size of all genome scaffolds totaled 957.40 Mb (Figure 1). The rate of genome length covered by these scaffolds was a noteworthy 96.32%. Notably, the final assembled size was slightly larger than initially predicted by the genome survey—a typical occurrence when using CCS data for assembly, especially in cases of high genetic variability. There was also a minor increase in the GC content to 34.77%, which, although minimal, marked an improvement over the earlier draft assembly. The scaffold N50 and N90 values were calculated to be 221.49 Mb and 81.12 Mb, respectively. These metrics further affirm the effectiveness and precision of our assembly approach, enriching our understanding of the *Z. transpicula* genome.

To illustrate the chromosomal arrangements, we performed collinearity analysis across four locust genomes (*Z. transpicula*, *E. oculatus*, *L. migratoria*, and *S. gregaria*) and uncovered a highly conserved syntenic relationship between the *Z. transpicula* and *E. oculatus* chromosomes (Supplementary Figure S1). Based on this observation, combined with previous reports on the sex chromosome of *E. oculatus* (6th longest, CM067381.1), we inferred that chromosome 5 (ZT5) represents the sex chromosome in *Z. transpicula*. Furthermore, by mapping the HiFi sequences used for genome assembly back to this assembly, the average coverage depth of ZT5 was only 34.03, significantly lower than other chromosomes, such as ZT6 at 44.31, while the genome-wide average coverage depth was 37.16 (Supplementary Figure S2). This result also supports the inference that ZT5 is the sex chromosome for the reduced coverage which aligns with the expectation of a heterogametic sex chromosome.

**Figure 2:**
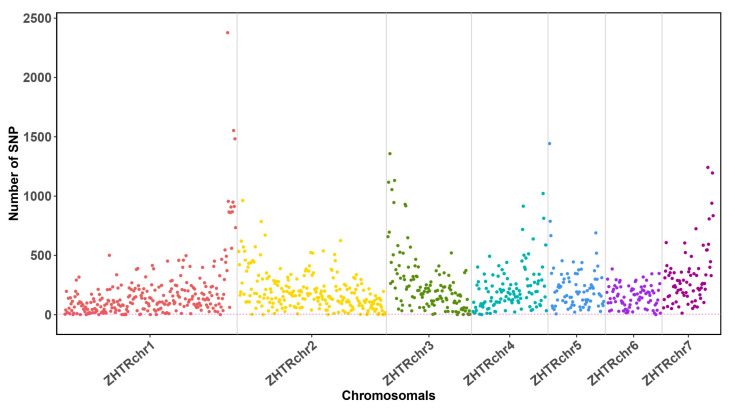
Manhattan plot illustrating variant distribution across the *Z. transpicula* Genome. Distinct colored dots denote various chromosomal locations, each corresponding to individual chromosomes. The *y*-axis represents the number of SNPs (Single Nucleotide Polymorphisms) within each sliding window (1 Mb).

To assess the quality of our chromosome-scale reference genome, we conducted an evaluation using Benchmarking Universal Single-Copy Orthologs (BUSCO). The analysis revealed remarkable gene completeness within the dataset under review (Figure 2). A standout finding is that an impressive 99.2% of the BUSCO groups assessed entirely matched the complete query sequences. Only a minor fraction (six genes) of the BUSCO groups were missing; 0.4% were found to be fragmented sequences. These results highlight our assembled genome sequence’s comprehensive coverage and excellent quality, confirming its reliability as a valuable resource for future genetic and genomic research. 

### 3.2. Low Genetic Heterozygosity Suggests a Small Effective Population Size in Z. transpicula

Our genomic assembly of *Z. transpicula* provides critical insights into the genetic diversity within this species, which is fundamental to assessing its evolutionary trajectory and conservation status. By aligning all next-generation sequencing (NGS) reads retrieved from another individual localized to the same geographic region against the newly assembled genome, we identified a meager count of 203,298 variants through the re-mapping process. The distribution of these variants across the chromosomes showed minimal disparity, with the majority of 1 Mb sliding windows accommodating fewer than 500 single nucleotide polymorphisms (SNPs) (Figure 2).

Considering the entirety of the compact *Z. transpicula* genome, virtually 970.40 Mb in size, the proportion of variants detected is substantially low (less than 0.02%), which was similarly diminutive (Figure 2). This low heterozygosity rate is even lower than that of the Giant Panda (*Ailuropoda melanoleuca*), which has heterozygosity of approximately 0.049% and is an iconic species under intense global conservation efforts known for its small effective population size and limited genetic diversity [31]. The paucity of genetic variation we report for *Z. transpicula* aligns uncannily with field observations indicating a constricted population size. Our genetic findings corroborate the premise that *Z. transpicula* harbors a critically low level of genetic diversity, thus categorizing it as an endangered species that requires immediate conservation attention.

**Figure 3:**
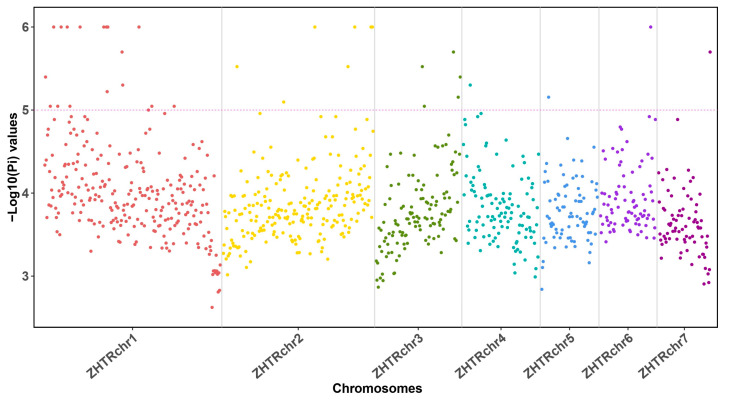
Manhattan plot illustrating nucleotide diversity across the *Z. transpicula* genome. Distinct colored dots denote various chromosomal locations, each corresponding to individual chromosomes. The *y*-axis represents the calculated −Log10 transformed Pi values within each sliding window (1 Mb).

Using an alternative measurement, we calculated nucleotide diversity (π) values for the *Z. transpicula* genome using the same 1 Mb sliding windows across the aligned re-sequencing data. The π values ranged from 2.62 to 6, with an average π of only 3.896 (Figure 3). Notably, over 70% of windows on chromosome 4 and over 50% on chromosome 5 had π values below 4. These π values are relatively lower than the typical threshold value of 5, indicating reduced nucleotide diversity (Figure 3). The generally low π values suggest that *Z. transpicula* has a reduced effective population size (Ne), reflecting a smaller pool of genetic diversity maintained within the species. The reduced Ne appears to have facilitated multiple selective sweeps across its genome, implying that *Z. transpicula* has undergone an apparent population bottleneck.

These findings, in conjunction with the low heterozygosity observed earlier, provide compelling evidence that *Z. transpicula* has experienced a significant decline in its population size, leading to a substantial reduction in genetic diversity. The limited genetic variation within this species raises concerns about its long-term viability and adaptability, underscoring the urgent need for conservation measures to safeguard its genetic diversity and ensure its survival in the face of environmental challenges.

### 3.3. Divergence Time Estimation and Gene Family Expansion/Construction Analysis

To provide insights into the putative evolutionary history and dynamics of gene family expansions and contractions across the order Orthoptera, we leveraged the *Z. transpicula* genome to construct the most comprehensive Bayesian genomic phylogenetic tree reported to date for this order, with all branches having a posterior probability of 1. This tree was based on a multiple sequence alignment of 1609 single-copy orthologs identified through OrthoFinder analysis, resulting in a 9.06 Mb protein alignment matrix.

**Figure 4:**
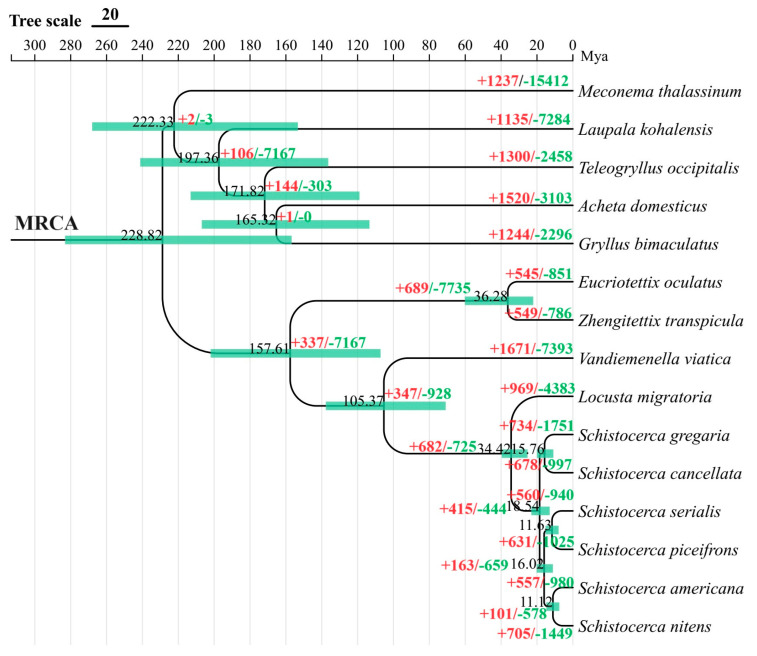
A phylogenetic tree constructed based on single-copy orthologous genes reported in 12 high-quality genomes from Orthoptera. The numbers on each branch indicate the predicted divergence time, and the green bars represent the predicted time intervals; the numbers at each node represent the gene family expansion and contraction numbers, in which red represents expansion and green represents contraction of gene families.

**Figure 5:**
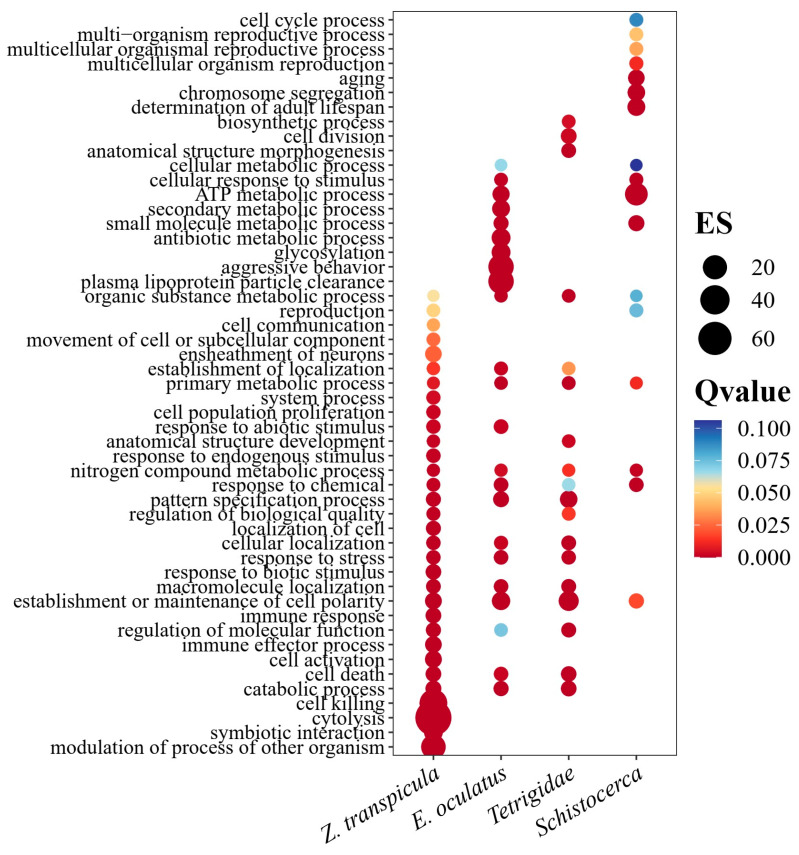
Gene Ontology (GO) enrichment analysis of expanded gene families in critical branches of interest—*Z. transpicula*, *E. oculatus*, Tetrigidae, and *Schistocerca*. The horizontal axis shows the class names, and the vertical axis shows the GO terms. Circle size represents the enrichment score (ES), and color indicates the Q-value (adjusted *p*-value), with red to blue corresponding to low to high values.

Our genomic study solidly confirmed our understanding of phylogenomic relationships within the superfamilies/families, supporting the monophyly of a group that uniquely encompasses *Z. transpicula* and *E. oculatus* from the family Tetrigidae, *V. viatica* from the superfamily Eumastacoidea, and *L. migratoria* from the superfamily Acridoidea (Figure 4). Notably, Tetrigidae was revealed as a distinct, independently diverging lineage, affirming its basal position within the broader Locustodea clade. Our divergence time estimates provide a chronological context for the evolution of the pygmy grasshoppers, placing the split between *Z. transpicula* and its closest relative, *E. oculatus*, at approximately 36.28 million years ago, during the late Eocene epoch. Expanding this clade further establishes the pivotal representation of the Tetrigidae lineage, estimated to have branched off around 157.61 million years ago, in the Mesozoic era (Figure 5). Our findings align with the available fossil evidence for Tetrigidae [32], cementing the family’s status as an ancient lineage distantly apart from Acridoidea, with an evolutionary history extending over 150 million years [33,34].

Delving deeper, we identified gene family contractions and expansions throughout the evolutionary history of these species, coupled with Gene Ontology (GO) enrichment analysis, to elucidate functional adaptations across different taxonomic levels. For *Z. transpicula*, we observed 549 expanded gene families, of which 21 were significantly enriched (*p* < 0.05) (Figure 4). These families, associated with cytolysis, cell killing, catabolic processes, and cell death, reveal an evolutionary edge, endowing *Z. transpicula* with enhanced cellular maintenance and turnover capacities. Such expansions indicate increased resilience against cellular damage, which is particularly advantageous given its constrained ecological niche. Likewise, the expansion of gene families related to cell activation and immune effector processes reflects a complex, likely pathogen-informed immune repertoire (Figure 5). These genomic footprints may reflect selective pressures from the diverse or pathogen-rich environments that this species has adapted to over time.

Moreover, the observed gene family expansions were found to be associated with the regulation of molecule locomotion, immune response, and stress response, highlighting *Z. transpicula*’s adaptive versatility. These families play a crucial role in adapting to biotic and abiotic challenges, providing clear evidence of the organism’s evolutionary acumen. Further, gene diversification linked to anatomical structure development and cell population proliferation contributes to developmental resilience. Significantly, we documented notable growth in gene families regulating cellular localization and movement of cell components, implying improved cellular communication and motility mechanisms that are integral to complex developmental and reproductive processes (Figure 5). An expanded repertoire of gene families involved in nitrogen compound metabolism and organic substance metabolism suggests a broad metabolic capacity that could be tied to the species’ specialized moss diet, underlining a genetic commitment to niche adaptation that is critical for their ecological conservation strategy.

These findings reveal the intricate genomic adaptations that have shaped *Z. transpicula*’s evolutionary trajectory, enabling its survival and thriving in its unique ecological niche. The observed gene family expansions and functional enrichments provide insights into the species’ enhanced cellular maintenance, immune capabilities, stress response mechanisms, developmental resilience, and metabolic versatility, all of which contribute to its remarkable ability to adapt to environmental challenges and occupy a specialized ecological role.

Compared to the more widespread *E. oculatus*, *Z. transpicula* exhibits several distinct evolutionary traits. In *E. oculatus*, 22 out of 545 distinct gene families were significantly expanded, particularly those involved in lipid metabolism, reflected in genes associated with plasma lipoprotein particle clearance (Figure 4). This process is vital for maintaining cellular homeostasis and energy storage, relevant to the agile life history of this species. Genomic evidence pointed to robust cellular machinery dedicated to establishing and maintaining cell polarity, possibly linked to the physical manifestation of observed aggressive behaviors and indicating a cellular basis for complex behavioral traits. Across the metabolic spectrum, our results highlighted considerable resources devoted to catabolic pathways, with genes mediating diverse biochemical conversions, including glycosylation and antibiotic degradation. This metabolic versatility reflects *E. oculatus*’ capacity for adaptive chemical and stress responses across ecological contexts. Supporting these findings, an examination revealed a robust framework for small molecule and organic substance metabolism, enhancing the efficacy in secondary metabolite production, ATP generation, and macromolecule localization and transport (Figure 5). This illuminates the bioenergetic and structural sophistication of *E. oculatus* at the cellular level.

Moreover, at the Tetrigidae family level, we observed similar expansion in 13 (a total of 689) gene families involved in the pattern specification process, indicating potential adaptive benefits in developmental precision (Figure 4). The diversification of these families reflects an evolutionary emphasis on accurate, regulated development that is essential for these pygmy grasshoppers inhabiting specialized niches (Figure 5). These families, linked to both catabolic and primary metabolic processes, reflect the necessity to efficiently manage energy resources, with expansions signaling robust metabolic capacity to derive energy through breaking down complex organic substances and sustaining the unique dietary habits of moss-feeding Tetrigidae. The regulation of molecular function and related biological quality within Tetrigidae also showed enrichment, suggesting enhanced fine-tuning of molecular interactions and adaptation to varying conditions.

Notably, gene families associated with anatomical structure morphogenesis and development were expanded, reflecting an evolutionary trajectory favoring versatile structural adaptations. Furthermore, expansions in cell death gene families indicate an intricate balance between proliferation and death that maintains healthy tissues and stress response. In response to cellular and environmental stimuli, Tetrigidae genomes showed amplifications in cellular and macromolecule localization families, reflecting a high degree of organization and transport efficiency critical to supporting cellular function across diverse conditions. Expansions in nitrogen compound metabolism may indicate specialized pathways to handle waste and assimilate nitrogen, which is crucial for survival in nitrogen-limited ecosystems (Figure 5).

These comparative analyses reveal the distinct evolutionary strategies and adaptations exhibited by *Z. transpicula*, *E. oculatus*, and the Tetrigidae family as a whole. The observed gene family expansions and functional enrichments highlight the diverse cellular processes, metabolic pathways, developmental mechanisms, and structural adaptations that have enabled these species to thrive in their respective ecological niches, reflecting the remarkable evolutionary versatility and resilience of these organisms.

Additionally, we compared differences in expanded gene families between the Tetrigidae and the desert locusts. The *Schistocerca* genomes exhibit a dense array of genes involved in small molecule metabolism, signaling robust cellular machinery adept at processing biochemicals. These are complemented by genes responsive to chemical stimuli, which may explain the locust’s ability to swiftly adapt to cues indicating nutrient availability or toxins (Figure 5). A notable finding is the association of annotated genes with adult lifespan, chromosome segregation, and aging, potentially elucidating life cycle dynamics and longevity across ecological contexts. The underlying molecular mechanisms governing cellular stimulus responses further reveal the locust’s ability to navigate its environment and maintain homeostasis under stress effectively. Nitrogen compound metabolism, crucial for synthesis and detoxification, is another pronounced genomic feature. The representation of primary metabolic processes showcases capabilities for fundamental biosynthetic and catabolic activities that are essential for survival and proliferation (Figure 5).

In *Schistocerca*, a substantial portion of expanded gene families is devoted to reproduction-related activities, including multicellular organism reproduction, cell polarity, reproductive processes, and multi-organism reproduction (Figure 5). This reflects a complex reproductive strategy potentially associated with the locust’s gregarious, swarming nature, aligning with the need for rapid population surges during swarming events that generate cascading ecological impacts. The diversity of genes involved in organic substance metabolism indicates extensive adaptability to various substrates, beneficial during swarming across diverse landscapes. Furthermore, identified cell cycle and metabolism genes suggest robust cell division and energy management, fundamental to the locust’s vigor, growth, and maintenance.

### 3.4. Divergent Evolution of the CYP305m2 Gene in Tetrigidae and Acrididae

Beyond variations in gene number, sequence variations in essential single-copy genes can directly contribute to functional differences among species. In locusts, the CYP305m2 gene is essential for controlling the production of phenylacetonitrile, an olfactory warning compound that can be further synthesized into hydrogen cyanide in gregarious locusts, serving to prevent cannibalism and defend against predators [35,36].

Based on the reported CYP305m2 sequence (QAU20957, 504aa), we screened for its homologs from currently available orthopteran genomes to analyze the sequence variations and infer their evolutionary implications, including the phenotypic differences between Tetrigidae and Acrididae. Out of 15 genomes used in the previous evolutionary analysis, we only identified 13 CYP305m2 homologs, with the absence of the gene in *M. thalassinum* and *A. domesticus*. In *G. bimaculatus*, *T. occipitalis*, and *V. viatica*, the gene sequences were incomplete, lacking critical elements such as promoters and terminators, suggesting that they are likely non-functional genomic fragments.

**Figure 6:**
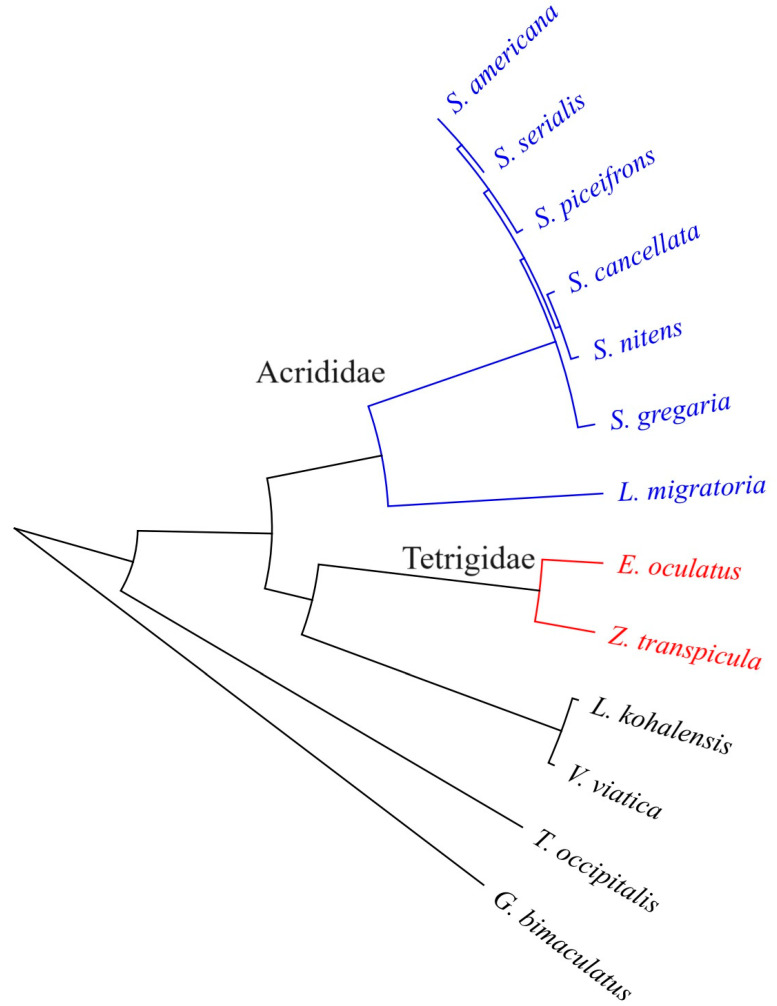
Phylogenetic relationships of CYP305m2 genes in 13 Orthoptera genomes. The unrooted tree was constructed using the UPGMA method based on an alignment of 13 CYP305m2 homologous sequences identified from 13 orthopteran genomes. Branches are color-coded by family—blue for Acrididae and red for Tetrigidae.

Through homology searches, we only found CYP305m2 gene homologs in the genomes of pygmy grasshoppers (Tetrigidae) and locusts (Acrididae), confirming the close phylogenetic relationship between these two families. The emergence of this vital gene was likely inherited from their common ancestor, with subsequent divergent neo-functionalization occurring since their evolutionary split. As intuitively shown in the gene clustering tree (Figure 6), the two Tetrigidae sequences clustered into one branch, while the Acrididae sequences clustered into another. We observed high cluster-specific sequence heterogeneity between these two families, implying that the CYP305m2 gene sequence is associated with their divergent evolutionary histories, leading to distinct adaptive advantages. Consistent with this view, and together with the absence of the gene in some species, these results further highlight the importance of CYP305m2, as its presence, absence, and sequence variations are closely related to the physiological ecology, species population dynamics, and swarming behavior, as well as pest occurrence and defense mechanisms in Orthoptera. 

**Figure 7:**
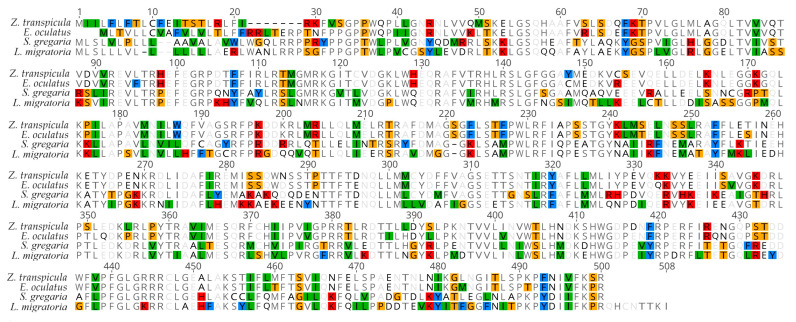
Protein sequence alignment of CYP305m2 homologs from *Z. transpicula*, *E. oculatus*, *L. migratoria*, and *S. gregaria*. All disagreements to consensus bases are highlighted using MacClade software’s (v4.06) color style.

A comparison of sequences revealed high similarity among Acrididae sequences, with pairwise identity as high as 98.0% among six *Schistocerca* species and 89.8% for *L. migratoria*. In contrast, the pairwise identity between *Z. transpicula* and *E. oculatus* was 88.9%, but the similarity dropped to 62.2% when compared to *L. migratoria* and *S. gregaria*, with only 221 identical sites accounting for less than 50% (Figure 7). The CYP305m2 sequence of *Z. transpicula*, which we report here for the first time, clusters with that of *E. oculatus*, suggesting a unique version of the gene evolved in Tetrigidae due to distinct selective pressures. The alignment of these two types revealed potential structural variations, suggesting that they have distinct functions which may have arisen due to different adaptive features.

In the N-terminal region, *L. migratoria* has approximately 10 more amino acids compared to *Z. transpicula* and *E. oculatus*, which may lead to minor differences in protein structure. There is a considerable variation in sequence length in the C-terminal region, implying frequent recombination in this region during evolution. The middle region of the sequence is relatively conserved, with some scattered amino acid substitutions (Figure 7). This indicates that the sequence divergence between Tetrigidae and Acrididae is substantial, and the two types of homologs likely contribute to the phenotypic differences observed between these two families.

## 4. Discussion

In this study, we endeavored to advance the genomic landscape of orthopteran insects by delivering a high-quality, chromosome-level reference genome assembly of the Chinese endemic pygmy grasshopper species, *Z. transpicula*. Our seminal work fills the substantial void in this taxon’s genomic data and establishes an essential platform for future biological and evolutionary explorations within the Tetrigidae family.

Our genome assembly, spanning an impressive 970.40 Mb with a scaffold N50 of 221.49 Mb, exemplifies data continuity and completeness. Remarkably, over 96% coverage of the estimated genome size was achieved, encapsulating the vast majority of *Z. transpicula*’s genetic repertoire. This sets a new standard within orthopteran genomics, outpacing many of the previously published insect genomes in quality [37,38]. Gene completeness, as highlighted by our BUSCO analysis with 99.2% of orthologs fully matched, further corroborates the thoroughness of our approach, reaffirming the representativeness of our gene space assembly. Notably, we successfully anchored the 54 longest contigs onto seven pseudo-chromosomes, aligning with the stable karyotype of 2n (♂) = 12 + X0 = 13 chromosomes in this group [39]. The collinearity analysis revealed a highly conserved syntenic relationship between the *Z. transpicula* and *E. oculatus* chromosomes, allowing us to infer that ZT5 represents the sex chromosome in *Z. transpicula* based on prior knowledge of the *E. oculatus* sex chromosome. This finding provides valuable insights into the chromosomal evolution and genomic architecture of pygmy grasshoppers.

Notably, the significantly lower average coverage depth observed for ZT5 compared to other chromosomes and the genome-wide average further corroborates our inference that ZT5 is the sex chromosome. This reduced coverage depth can be attributed to the inherent heterogametic nature of sex chromosomes. In pygmy grasshoppers, females carry only one X sex chromosome. Consequently, the coverage depth of the heterogametic sex chromosome is expected to be lower compared to autosomes, as it is present in a single copy within the sequenced pool. The observed reduced coverage of ZT5 is consistent with this phenomenon, strongly supporting our conclusion that it represents the sex chromosome in the *Z. transpicula* genome.

The genotypic insights gleaned from our re-mapping genomic analysis illuminate the dire conservation status of *Z. transpicula*. Despite repeated sampling efforts, this species was rarely encountered, with only a few individuals collected over several months. An alarmingly low heterozygosity rate of 0.02% suggests a genetic bottleneck, likely due to the species’ narrow habitat range on Mt. Shiwandashan, limited population size, and specialized niche within humid mossy forests [40,41,42]. Compared with other endangered species, such a low heterozygosity rate is lower than that of the giant panda (*Ailuropoda melanoleuca*) and comparable to that of the Orca (*Orcinus orca*) [31]. Theoretical interpretations align with these genomic observations, where exceedingly low π values and sparse SNP presence may indicate considerable genetic drift and selective sweeps. As genetic diversity is depleted, *Z. transpicula* spirals closer to an extinction vortex. Given these findings, conservation measures must be swiftly enacted to protect and restore this unique taxon’s diminishing genetic diversity and habitat.

Analyzing the evolution of *Z. transpicula* from a phylogenomic perspective, we delineate its evolutionary trajectory, which appears intertwined with significant climatic events. The estimated divergence from its closest relative, *E. oculatus*, roughly 36 million years ago, aligns with significant environmental shifts corresponding to the onset of Antarctic cooling, suggesting that pygmy grasshoppers may have adapted to these changes, leading to the diverse and alpine distributions that we observe today. This narrative is supported by the genomic divergence dating back to over 150 million years, potentially driven by early continental drift events during the Jurassic, which laid the groundwork for the current ecological diversity [32,43,44].

Our subsequent investigations into genomic novelty uncovered expansions in gene families associated with cellular processes, metabolism, and stress response, underscoring the intense selective pressures for survival and functional optimization within *Z. transpicula*. This genomic adaptation to environmental niches further differentiates pygmy grasshoppers from the more generalized locusts, offering insights into the processes driving the evolutionary specializations of these orthopterans [4,38,45]. Consistent with previous molecular genetic studies of pygmy grasshoppers and locusts using transcriptomes and genomes, the functions of these genes largely agree with these studies, such as pigment, immunity, and insecticide resistance, reflecting the genetic uniqueness of Tetrigidae [4,8,38,45]. Combining all these observations, we bolster the hypothesis that the expanded gene families within *Z. transpicula* are instrumental in forging its specialized ecological role. Immunity-related and stress-response genes are likely a response to pathogen pressures in humid, mossy forests, while genes associated with detoxification processes may reflect Tetrigidae’s need to manage diverse dietary and environmental toxins.

Furthermore, we tackle the ecological disparities between pygmy grasshoppers and the more destructive migrating swarming locusts, focusing on the critical CYP305m2 gene. While found in both lineages, our comparative analysis reveals substantial sequence deviation among these two families, pointing to potential neofunctionalizations. The altered CYP305m2 in swarming locusts, with only 221 (35%) amino acid similarity to that of Tetrigidae, indicates adaptive divergence, feasibly reflecting biochemical shifts that mitigate gregarization propensities. Such discoveries might explain why only the Acrididae are noxious pests and prompt deeper inquiry into the molecular factors contributing to the nuances of ecological interactions within the Orthoptera [35,44,46,47,48].

In summary, as presented in this work, the genomic narrative of *Z. transpicula* enriches our taxonomic knowledge and emphasizes the urgency for conservation, the power of climatic forces in shaping evolution, and the intricacies of gene function adaptation. Our study stands as a call to action for intensified research and protective measures for endangered species whose genetic and ecological distinctiveness are yet to be fully understood. The genetic foundation underlying the unique ecological features of Tetrigidae has been elucidated, providing valuable insights into the evolutionary processes that have shaped this remarkable group of organisms.

## Data Availability

The assembled genome from this study has been submitted to NCBI’s BioProject database under accession number PRJNA1035776. The genome assembly and the annotation gff3 file can be also accessed on the Zenodo database through the link of https://zenodo.org/records/10171548, accessed on 21 November 2023.

## Supplementary Materials

The following supporting information can be downloaded at: https://www.mdpi.com/article/10.3390/insects15040223/s1, Supplementary Figure S1: Genomic collinear plot among four selected genomes of *Z. transpicula*, *E. oculatus*, *L. migratoria*, and *S. gregaria*. Supplementary Figure S2: Coverage distribution of HiFi sequences used for genome assembly across different chromosomes in *Z. transpicula*. Supplementary Table S1: Genome assembly and chromosome anchoring result parameters statistics. Supplementary Table S2: Statistics for Protein-coding gene models. Supplementary Table S3: Gene annotations using Interproscan. Supplementary Table S4: Statistics for BUSCO analysis, both genome and protein. Supplementary Table S5: List of species used in the comparative analyses.
