# Supplementary figures and images for: Chromosomal-Level Reference Genome for the Chinese Endemic Pygmy Grasshopper, Zhengitettix transpicula, Sheds Light on Tetrigidae Evolution and Advancing Conservation Efforts
Source: Insects. 2024 Mar 25;15(4):223. doi: 10.3390/insects15040223 (PMC11049975; doi:10.3390/insects15040223)

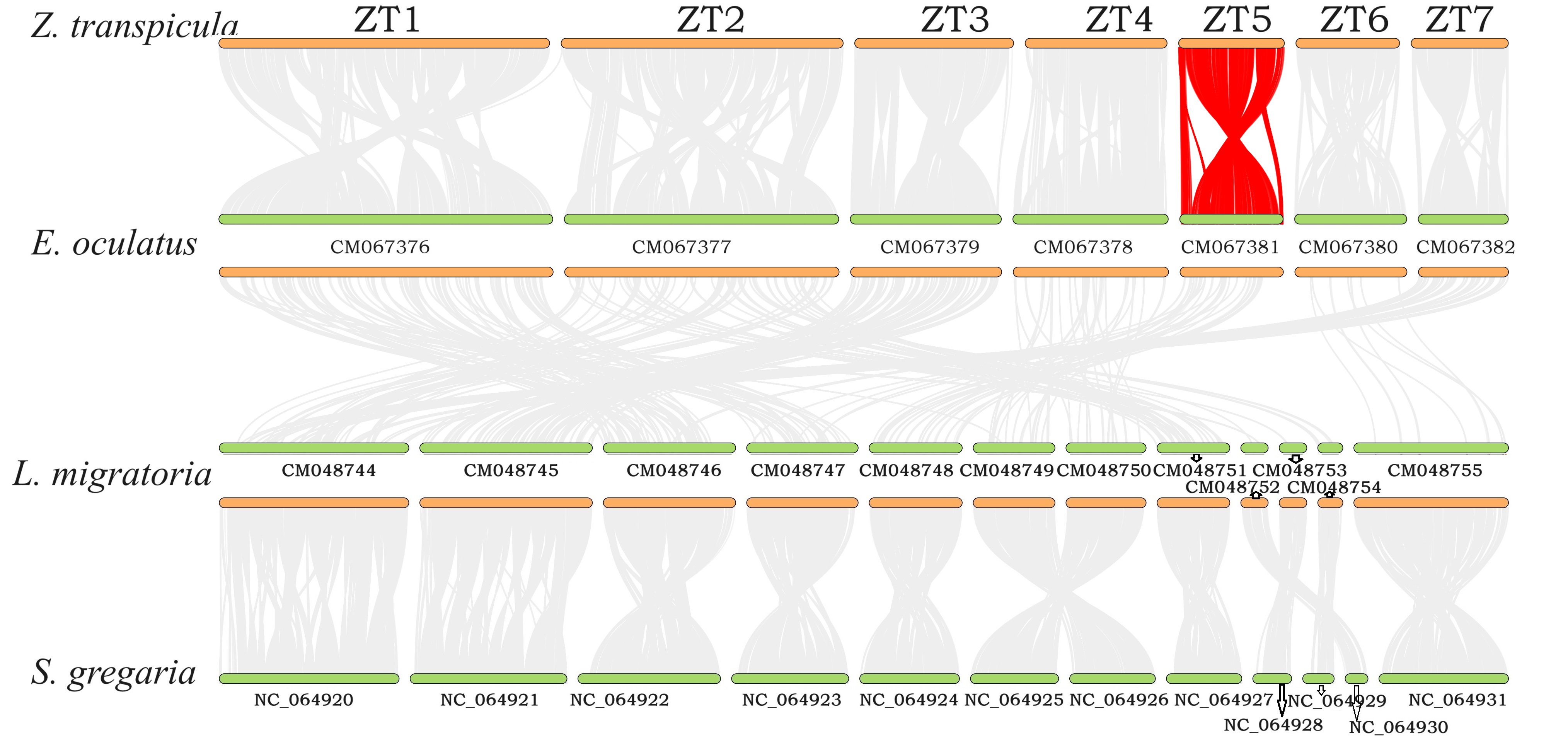

Supplement: Supplementary file 1 [file insects-15-00223-s001.zip › Supplement Figure S1.jpg]

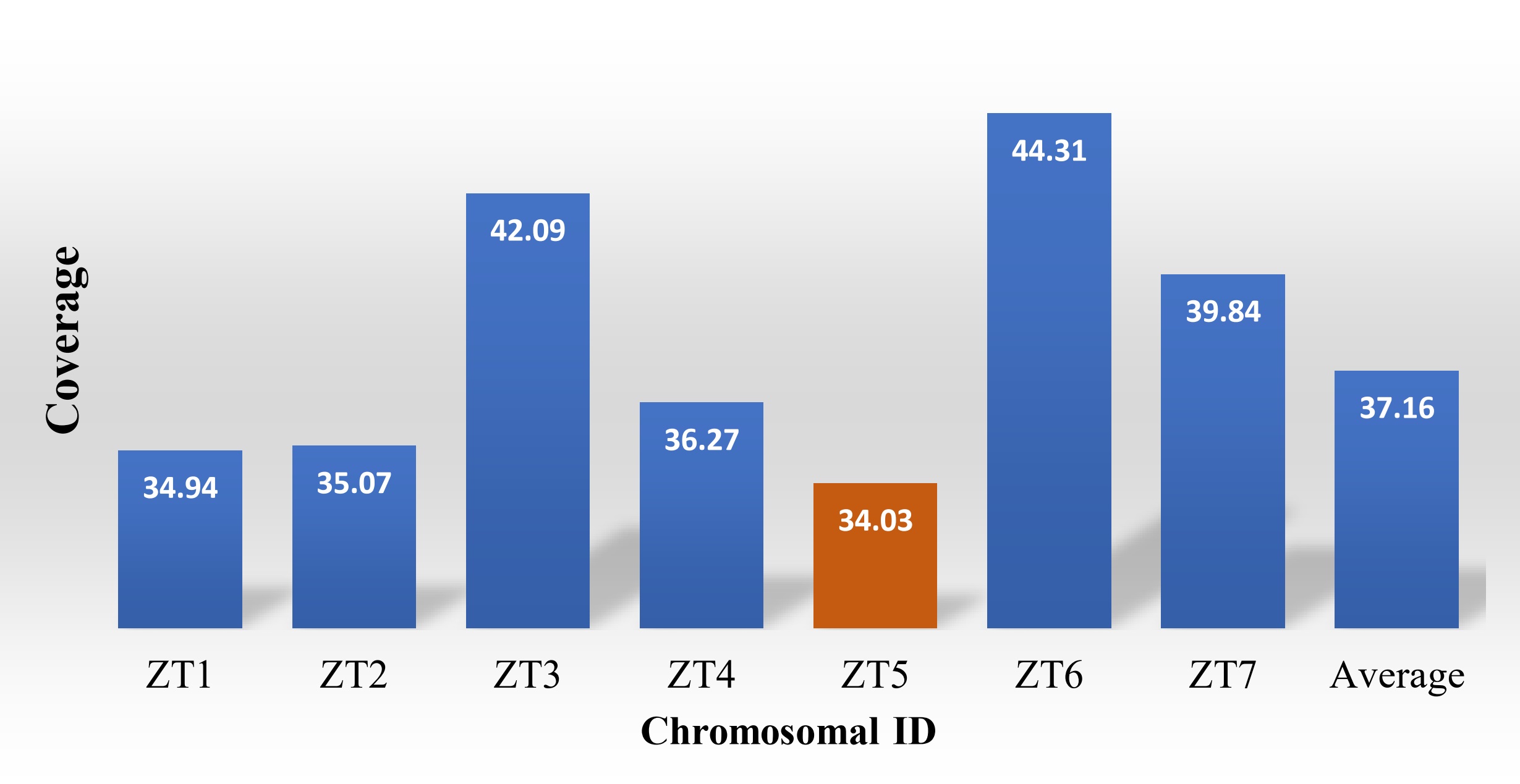

Supplement: Supplementary file 1 [file insects-15-00223-s001.zip › Supplement Figure S2.jpg]
